# The Throat and Its Diseases

**Published:** 1887-09

**Authors:** 


*The Throat and its Diseases.* By LENNOX BROWNE, F.R.C.S.E. Pp. 614. 2nd edition. London: BAILLIÈRE, TINDALL, and COX. 1887.

As compared with the first edition, which has been out of print eight years, this second one is practically a new work. In its present form the book may be regarded as a complete exposition of the Diseases of the Throat from a pathological as well as from a clinical and therapeutical standpoint. The arrangement and subdivision of matter are admirable. A few introductory chapters are given on the Anatomy, and the examination of parts; on General Semeiology and Therapeutics, and on General Pathology. Then follow careful systematic descriptions of the Diseases of the Pharynx, the Uvula, the Tonsils, the Larynx, and the Nasal Passages. While the Pathology, the Etiology, and the Semeiology are treated with sufficient fulness, and with admirable balance and judgment, most praise must be bestowed on the parts devoted to treatment. Here the author speaks out definitely and clearly from convictions begotten of a large and well-utilised experience. And everywhere throughout the work there are evidences of extensive study, close reasoning, sound judgment, and skilful practice. Clearly the work must at once take a leading, if not *the* leading, position among works in the English language on Diseases of the Throat.

Not the least valuable part of the work is its illustrations. Nearly all of them are the author's own work, either drawn on wood, or reproduced by photo-gravure from pen-and-ink sketches, or drawn on stone. Fifteen plates of admirable lithographs, representing in a lifelike manner the most varied diseases, provide the student with a wealth of illustration which is positively luxurious. And these plates are so bound in the volume that any one of them can be unfolded and laid open to view while the letterpress is being perused. Indeed, the whole get-up of the work is such as to reflect the greatest credit on all concerned in its production. It is easy to foretell for it an extensive sale and a wide usefulness.

